# Complete Genome Sequence of *Southern tomato virus* Naturally Infecting Tomatoes in Bangladesh

**DOI:** 10.1128/genomeA.01522-15

**Published:** 2015-12-31

**Authors:** Chellappan Padmanabhan, Yi Zheng, Rugang Li, Zhangjun Fei, Kai-Shu Ling

**Affiliations:** a USDA–Agricultural Research Service, U.S. Vegetable Laboratory, Charleston, South Carolina, USA; b Boyce Thompson Institute for Plant Research, Cornell University, Ithaca, New York, USA; c USDA–Agricultural Research Service, Robert W. Holley Center for Agriculture and Health, Ithaca, New York, USA

## Abstract

The complete genome sequence of a *Southern tomato virus* (STV) isolate on tomato plants in a seed production field in Bangladesh was obtained for the first time using next-generation sequencing. The identified isolate, STV_BD-13, shares a high degree of sequence identity (99%) with several known STV isolates worldwide.

## GENOME ANNOUNCEMENT

*Southern tomato virus* (STV) is a double-stranded RNA (dsRNA) virus with a small genome of approximately 3.5 kb containing two overlapping open reading frames (ORFs). Based on its dsRNA genome and the pattern of genome structure, STV was classified in the genus *Amalgavirus* of the family *Amalgaviridae* (1). STV has been regarded as a causal agent of disease in tomato (1). However, its biological effect on infected plants is in need of further investigation. Since its first discovery on tomato plants in Mexico and the United States (1), STV has also been identified in France (2), Spain (3), and most recently in China (4). In most of these cases, STV was presented in field samples with a mixed infection with other viruses. In France, STV was identified along with *Tobacco mosaic virus* (TMV) and *Potato virus Y* (PVY) (2). In Spain, STV was also in a coinfection with *Pepino mosaic virus* (PepMV), *Tomato spotted wilt virus* (TSWV), and *Tomato yellow leaf curl virus* (TYLCV) (3). The recently identified STV from China was also in a mixed infection with TYLCV, *Cucumber mosaic virus* (CMV), and *Tomato chlorosis virus* (4). In summer 2013, tomato plants in a seed production field in Bangladesh were observed to exhibit a high incidence of virus-like disease symptoms, including severe mosaic, epinasty, and yellow stunting disorders. To determine the causal agent(s), small RNA (sRNA) deep-sequencing technology (5) was employed for virus identification. Total RNA was prepared on a bulked tomato sample using TRIzol reagent (Life-Technology, USA). An sRNA library was prepared as described (6) and sequenced using an Illumina HiSeq 2000. To identify possible viruses, sRNA sequences were first assembled into contigs using the previously established bioinformatics pipeline after subtraction of host tomato-derived sRNAs (7). From the preliminary sequence assemblies and analyses, in addition to the full genome of STV in a single contig, two other viruses (PVY and CMV) were also identified (data not shown). For STV, its sequence was verified with Sanger sequencing using overlapping PCR products. The complete genome for the Bangladesh isolate STV_BD-13 comprised 3,438 nucleotides encoding only two ORFs. The 5′-proximal ORF encoded a 378–amino acid (aa) peptide representing a potential coat protein (p42). The second ORF contained typical motifs for an RNA-dependent RNA polymerase (1,063 aa) and was likely expressed via a +1 ribosomal frameshift, in agreement with the earlier report (1). BLASTn searches to the NCBI databases revealed that STV_BD-13 shares 99% sequence identity with other STV isolates in Mexico (EF442780), the United States (EU413670), France (KC333078), and China (KT438549). To our knowledge, this is the first report of STV (STV_BD-13) in Bangladesh and the second case in Asia, after only China (4). Such broader distribution of STV has caused concerns to the tomato industry. Experiments should be conducted to determine the impact of STV on tomato productions and its effect through seed transmission.

### Nucleotide sequence accession number.

The genome sequence of *Southern tomato virus* isolate STV_BD-13 has been deposited in GenBank under the accession number KT634055.

## References

[1] Sabanadzovic S, Valverde RA, Brown JK, Martin RR, Tzanetakis IE 2009 Southern tomato virus: the link between the families *Totiviridae* and *Partitiviridae*. Virus Res 140:130–137. doi:10.1016/j.virusres.2008.11.018. 19118586

[2] Candresse T, Marais A, Faure C 2013 First report of Southern tomato virus on tomatoes in southwest France. Plant Dis 97:1124. doi:10.1094/PDIS-01-13-0017-PDN. 30722473

[3] Verbeek M, Dullemans AM, Espino A, Botella M, Alfaro-Fernández A, Font MI 2015 First report of Southern tomato virus in tomato in the Canary Islands, Spain. J Plant Pathol 97:392. doi:10.4454/JPP.V97I2.038.

[4] Padmanabhan C, Zheng Y, Li R, Sun S, Zhang D, Liu Y, Fei Z, Ling K 2015 Complete genome sequence of *Southern tomato virus* identified in China using next-generation sequencing. Genome Announc 3(5):e01226-15. doi:10.1128/genomeA.01226-15. PMC4616180 26494671

[5] Kreuze JF, Perez A, Untiveros M, Quispe D, Fuentes S, Barker I, Simon R 2009 Complete viral genome sequence and discovery of novel viruses by deep sequencing of small RNAs: a generic method for diagnosis, discovery and sequencing of viruses. Virology 388:1–7. doi:10.1016/j.virol.2009.03.024. 19394993

[6] Chen Y, Zheng Y, Liu B, Zhong S, Giovannoni J, Fei Z 2012 A cost-effective method for Illumina small RNA-Seq library preparation using T4 RNA ligase 1 adenylated adapters. Plant Methods 8:41. doi:10.1186/1746-4811-8-41. 22995534 PMC3462708

[7] Li R, Gao S, Hernandez AG, Wechter WP, Fei Z, Ling K 2012 Deep sequencing of small RNAs in tomato for virus and viroid identification and strain differentiation. PLoS One 7:e37127. doi:10.1371/journal.pone.0037127. 22623984 PMC3356388

